# Evolutionary Models for the Diversification of Placental Mammals Across the KPg Boundary

**DOI:** 10.3389/fgene.2019.01241

**Published:** 2019-11-29

**Authors:** Mark S. Springer, Nicole M. Foley, Peggy L. Brady, John Gatesy, William J. Murphy

**Affiliations:** ^1^Department of Evolution, Ecology, and Evolutionary Biology, University of California, Riverside, Riverside, CA, United States; ^2^Department of Veterinary Integrative Biosciences, Texas A&M University, College Station, TX, United States; ^3^Division of Vertebrate Zoology, American Museum of Natural History, New York, NY, United States

**Keywords:** KPg boundary, placental radiation, relaxed clocks, timetrees, tip dating

## Abstract

Deciphering the timing of the placental mammal radiation is a longstanding problem in evolutionary biology, but consensus on the tempo and mode of placental diversification remains elusive. Nevertheless, an accurate timetree is essential for understanding the role of important events in Earth history (e.g., Cretaceous Terrestrial Revolution, KPg mass extinction) in promoting the taxonomic and ecomorphological diversification of Placentalia. Archibald and Deutschman described three competing models for the diversification of placental mammals, which are the *Explosive*, *Long Fuse*, and *Short Fuse Models*. More recently, the *Soft Explosive Model* and *Trans-KPg Model* have emerged as additional hypotheses for the placental radiation. Here, we review molecular and paleontological evidence for each of these five models including the identification of general problems that can negatively impact divergence time estimates. The *Long Fuse Model* has received more support from relaxed clock studies than any of the other models, but this model is not supported by morphological cladistic studies that position Cretaceous eutherians outside of crown Placentalia. At the same time, morphological cladistics has a poor track record of reconstructing higher-level relationships among the orders of placental mammals including the results of new pseudoextinction analyses that we performed on the largest available morphological data set for mammals (4,541 characters). We also examine the strengths and weaknesses of different timetree methods (node dating, tip dating, and fossilized birth-death dating) that may now be applied to estimate the timing of the placental radiation. While new methods such as tip dating are promising, they also have problems that must be addressed if these methods are to effectively discriminate among competing hypotheses for placental diversification. Finally, we discuss the complexities of timetree estimation when the signal of speciation times is impacted by incomplete lineage sorting (ILS) and hybridization. Not accounting for ILS results in dates that are older than speciation events. Hybridization, in turn, can result in dates than are younger or older than speciation dates. Disregarding this potential variation in "gene" history across the genome can distort phylogenetic branch lengths and divergence estimates when multiple unlinked genomic loci are combined together in a timetree analysis.

## Introduction

Placentalia is the crown clade of eutherian mammals and includes 18–19 different orders with living representatives plus other major groups that are entirely extinct (e.g., Meridiungulata, Creodonta, Dinocerata, Mesonychia, Embrithopoda, Desmostylia, and Leptictida). Resolving the timing of the placental mammal radiation, both between orders and within orders, is a longstanding problem in evolutionary biology (Szalay, 1968; Gingerich, 1977; Young, 1981; Springer et al., 2003; Meredith et al., 2011; dos Reis et al., 2012; Halliday et al., 2019). Elucidation of the timing of this radiation has important implications for understanding the role of the KPg mass extinction in promoting the radiation of placental mammals.

The traditional view based on paleontology is that placental mammals began to diversity near the end of the Cretaceous, but with the bulk of the interordinal radiation occurring after the KPg mass extinction ∼66 Ma (e.g., Szalay, 1968; Gingerich, 1977; Young, 1981; Carroll, 1988). Nevertheless, some paleontologists have allowed for the possibility that incipient cladogenesis among extant placental lineages may have occurred as far back as 85–80 Ma (Szalay, 1968; Young, 1981; Carroll, 1988) or even as far back as the Early Cretaceous. For example, McKenna and Bell (1997) included 22 genera from the Late Cretaceous and one genus from the Early Cretaceous in the crown-group Placentalia.

Early studies based on molecular data employed strict or local molecular clocks (Dickerson, 1971; Li et al., 1990), sometimes with culling of genes for which a constant rate of evolution was rejected by likelihood ratio tests and/or linearized tree tests (Hedges et al., 1996; Kumar and Hedges, 1998), or with adjustments for rate variation based on a reference taxon (Springer, 1997; Springer et al., 1997). Quartet dating (Rambaut and Bromham, 1998) allowed for limited rate variation under a 2-rate model and was also applied to early divergences in the placental radiation (Eizirik et al., 2001; Madsen et al., 2001; Murphy et al., 2001a; Scally et al., 2001). The general consensus of these studies was that most interordinal cladogenesis occurred prior to the KPg boundary. Moreover, many of these studies pushed back the estimate for the most recent common ancestor of Placentalia to ∼100 Ma or more (Li et al., 1990; Hedges et al., 1996; Kumar and Hedges, 1998; Eizirik et al., 2001; Madsen et al., 2001; Murphy et al., 2001b). Some of these studies also suggested that plate tectonic events could have been important drivers of the early placental radiation (Hedges et al., 1996; Springer et al., 1997; Eizirik et al., 2001; Murphy et al., 2001a; Murphy et al., 2001b; Scally et al., 2001; Wildman et al., 2007).

**Figure 1:**
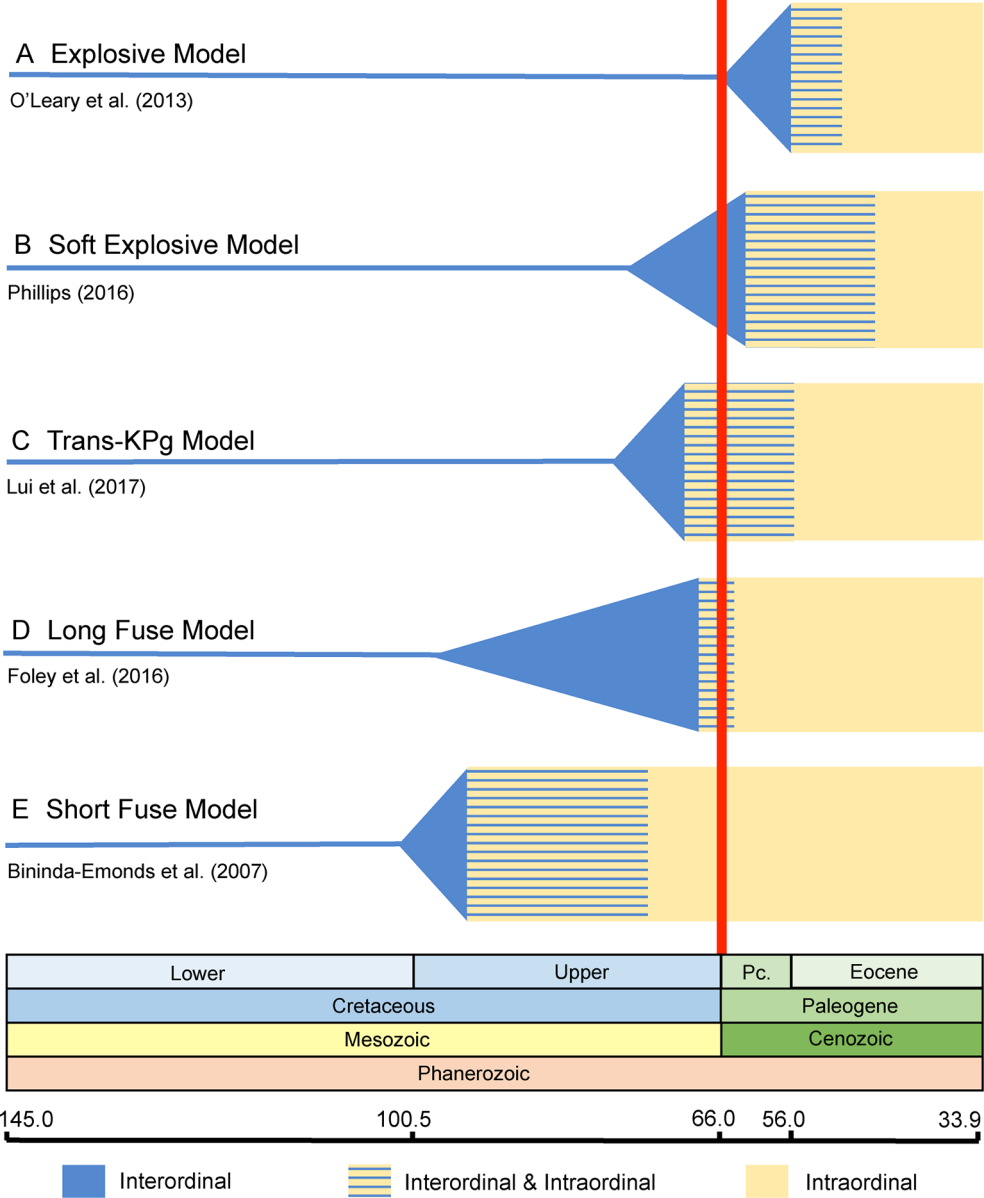
Graphical summary of the five competing models of diversification for placental mammals. Approximate dates that were used to illustrate each model are derived from representative studies as indicated in the figure. **(A)** *Explosive Model*. **(B)** *Soft Explosive Model*. **(C)** *Trans-KPg Model*. **(D)** *Long Fuse Model*. **(E)** *Short Fuse Model*. For the *Short Fuse Model*, some molecular estimates for the base of Placentalia are older than the date obtained by Bininda-Emonds et al. (2007), e.g., Kumar and Hedges (1998) obtained a date of ∼129 Ma.

Archibald and Deutschman (2001) proposed three competing models for the placental radiation based on published paleontological and molecular dating studies. These are the *Explosive, Long Fuse*, and *Short Fuse Models* of diversification (**Figure 1**). The *Explosive Model* corresponds to the widely held view among paleontologists that most or all of the placental radiation occurred after the KPg mass extinction. This model also suggests a fundamental role for the mass extinction in promoting the interordinal radiation of placental mammals. The *Short Fuse* and *Long Fuse Models*, in turn, emerged from molecular-based studies and posit deeper temporal roots for the placental radiation. In the *Long Fuse Model*, interordinal cladogenesis is primarily concentrated before the KPg boundary whereas intraordinal cladogenesis occurred after the end Cretaceous mass extinction. The *Short Fuse Model* posits even more ancient interordinal cladogenesis, in some cases as far back as the Jurassic, along with the beginnings of intraordinal cladogenesis in numerous orders in the Cretaceous. These three models have provided a useful framework for subsequent studies of the placental radiation.

Several developments near the turn of the millennium helped to shape the last ∼20 years of timetree studies on placental mammals. First, Thorne et al. (1998) developed a Bayesian Markov chain Monte Carlo (MCMC) method that allows each branch to have its own rate of evolution. Second, the traditional phylogeny for placental orders based on morphology (e.g., Szalay, 1977; Novacek, 1992) was overhauled by molecular studies that employed multigene data sets and improved models of sequence evolution. The results of these studies clustered placental orders into four major clades (Afrotheria, Xenarthra, Euarchontoglires, Laurasiatheria) with the additional clustering of Euarchontoglires and Laurasiatheria into Boreoeutheria (Springer and de Jong, 2001). Of these five clades only Xenarthra was recovered by previous morphological analyses. This overhaul began with the recognition of Afrotheria (Springer et al., 1997; Stanhope et al., 1998a; Stanhope et al., 1998b) and came to full fruition in multigene studies that provided robust support for the four major clades of placental mammals (Madsen et al., 2001; Murphy et al., 2001a; Murphy et al., 2001b; Scally et al., 2001; Waddell et al., 2001; Springer et al., 2004). All four major clades, as well as Boreoeutheria, have been corroborated by retroposon insertions (Nishihara et al., 2006; Möller-Krull et al., 2007; Nishihara et al., 2009). This thoroughly revised phylogeny for placental mammals also overturned previous molecular hypotheses based on mitogenomes (D’Erchia et al., 1996; Reyes et al., 2000; Arnason et al., 2002) and early analyses of nuclear genes with limited taxon sampling (e.g., Graur et al., 1991; Li et al., 1992; Graur et al., 1992) that positioned rodents or erinaceids (e.g., hedgehogs, moon rats) as the earliest branches of the placental tree. The root of the placental tree remains contentious, but is now centered on three competing hypotheses: Afrotheria versus Boreoeutheria + Xenarthra; Afrotheria + Xenarthra versus Boreoeutheria; and Xenarthra versus Afrotheria + Boreoeutheria (Scally et al., 2001; Murphy et al., 2007; Morgan et al., 2013; Romiguier et al., 2013; Tarver et al., 2016). Resolution of this uncertainty is important for dating the placental tree.

Most timetree studies based on well-corroborated molecular topologies have recovered the majority of interordinal divergences in the Cretaceous and are generally compatible with the *Long Fuse Model* (Springer et al., 2003; Delsuc et al., 2004; Springer et al., 2005; Murphy et al., 2007; Meredith et al., 2011; dos Reis et al., 2012; Tarver et al., 2016; Foley et al., 2016). An exception is the supertree analysis by Bininda-Emonds et al. (2007), which recovered even older divergence times that are generally compatible with the *Short Fuse Model*. By contrast, the authors of recent morphological cladistic studies have argued that their results provide renewed support for a strict version of the *Explosive Model* of diversification by positioning all Cretaceous taxa outside of Placentalia (Wible et al., 2007; Wible et al., 2009; O’Leary et al., 2013). For example, O’Leary et al. (2013) claimed that all interordinal cladogenesis occurred after the KPg mass extinction based on their combined analyses of morphology and molecules.

Recently, Phillips (2016) and Liu et al. (2017a) proposed new models of placental diversification that are intermediate between the *Long Fuse* and *Explosive Models*. Specifically, Phillips (2016) proposed the *Soft Explosive Model* and Liu et al. (2017a) proposed the *Trans-KPg Model* (**Figure 1**). These models are slight variations of the same theme, that interordinal diversification extended across the KPg and well into the Cenozoic, when most intraordinal diversification occurred, and bring the total number of models from three to five.

A common denominator of relaxed molecular clock analyses of the placental radiation is that they have generally relied on node-dating approaches that calibrate a rooted tree by constraining the age of one or more internal nodes (Springer et al., 2003; Delsuc et al., 2004; Springer et al., 2005; dos Reis et al., 2012; Emerling et al., 2015; Phillips, 2016; Foley et al., 2016; Tarver et al., 2016; Liu et al., 2017a). Node dating has recently come under scrutiny, perhaps most importantly because the maximum age and prior probability distribution for a calibrated node are subjective (Heath et al., 2014; Lee and Palci, 2015). Given this and other potential problems with node dating, alternative approaches for timetree inference have gained more traction. One popular method is tip dating (Pyron, 2011; Ronquist et al., 2012). This approach was originally developed for dating evolutionary trees of RNA viruses with samples that were taken at different years (Rambaut, 2000). Dated tips provide a unique source of information for estimating rates of evolution and time-scaling a tree (Lee and Palci, 2015). Tip dating of sequential samples of RNA viruses was co-opted for use with evolutionary trees that include fossil organisms. To achieve this goal, the RNA clock for sequentially sampled viruses has been replaced with a morphological clock for phenotypic characters that are scored for extinct and extant taxa (Lee and Palci, 2015). Tip dating can also take advantage of molecular matrices for extant taxa, in which case the term "total evidence dating" is sometimes used because the data sets contain both molecular and morphological characters (Lee and Palci, 2015). For convenience we use the term tip dating for the remainder of this paper. In tip dating, the molecular and morphological data matrices are simultaneously used to estimate the phylogenetic placement of fossils and calibrate the tree (Arcila et al., 2015). An additional advantage of tip dating is that all extinct species for a given clade can be included in analysis, rather than just the oldest fossil as in node dating.

In addition to tip dating, Heath et al. (2014) suggested a new method for timetree estimation that uses a single model for the speciation-extinction-fossilization process. This model is known as the fossilized birth–death model and has only four parameters (speciation rate, extinction rate, fossil recovery rate, proportion of sampled extant species) that require prior assumptions. Fossilized birth–death dating can be implemented with tip dating (Gavryushkina et al., 2017), but in its original incarnation (Heath et al., 2014) fossilized birth-death dating was performed with molecular data only. A more recent implementation of fossilized birth–death dating requires fossil ages and a set of trees, but does not require molecular data (Didier and Laurin, 2018).

An additional issue that affects the estimation of species divergence times with molecular data is that coalescence times for individual genes are expected to exceed speciation times. For segments of the genome that disagree with the species tree because of incomplete lineage sorting (ILS), coalescence times will always exceed speciation times (Angelis and dos Reis, 2015; dos Reis et al., 2016). ILS, also known as deep coalescence when viewed from the perspective of looking back in time, occurs when alleles fail to coalesce in the most recent common ancestor of two taxa and instead coalesce deeper in the gene tree. A consequence of ILS is that divergence times on gene trees will overestimate speciation times. However, even gene segments that agree with the species tree are expected to have coalescence times that exceed speciation times. The opposite pattern may occur when two taxa hybridize with each other. Specifically, gene flow between two taxa, either involving portions of the nuclear genome and/or the mitogenome, will result in a divergence time estimate for these taxa that is younger than the actual speciation time for the same taxa when the introgressed DNA regions are employed in timetree analyses. Recent studies suggest that extensive introgression has occurred in several mammalian clades (e.g., Li et al., 2016; Árnason et al., 2018; Palkopoulou et al., 2018; Li et al., 2019), so this issue deserves consideration in future timetree studies given that all current molecular clock estimation models assume no gene flow among species lineages.

Here, (1) we review the supporting arguments and shortcomings of each of the five models of placental diversification, including the identification of general problems that can negatively impact divergence time estimates; (2) examine the pros and cons of different timetree methods (node dating, tip dating, fossilized birth–death dating) that may now be applied to estimate the timing of the placental radiation; and (3) discuss the complexities of timetree estimation when the genetic signal for speciation times is complicated by the coalescence process and hybridization (Hallström and Janke, 2008).

## Review and Comparison of Models

### Explosive Model

The *Explosive Model* posits that the vast majority of placental cladogenesis, both interordinal and intraordinal, occurred near or after the KPg boundary (66 Ma) (**Figure 1A**) (Archibald and Deutschman, 2001). During the first ∼10 million years of the Cenozoic, diversification of terrestrial placental taxa occurred rapidly in response to available niche space vacated by non-avian dinosaurs (Carroll, 1997; O’Leary et al., 2013). Support for *Explosive Model* is derived from direct reading of the fossil record and also from trees derived from the analysis of morphological data that exclude all or most Mesozoic eutherians from crown Placentalia (Gingerich, 1977; Archibald and Deutschman, 2001; Gingerich et al., 2001; Wible et al., 2009; Goswami et al., 2011; O’Leary et al., 2013; Davies et al., 2017). Instead, most Mesozoic eutherians are positioned as stem placental lineages (Archibald and Deutschman, 2001); throughout the remainder of our discussion, we refer to extinct eutherians that are outside of Placentalia as "stem placentals." Recent versions of the *Explosive Model*, which are based on cladistic analyses of large morphological and combined data sets (Wible et al., 2007; Wible et al., 2009; O’Leary et al., 2013; Halliday, et al., 2016; Halliday et al., 2017; Halliday et al., 2019), suggest an extreme version of the *Explosive Model* that is consistent with just a single placental ancestor crossing the KPg boundary.

A literal reading of the fossil record indicates that there is a striking increase in the abundance of extinct eutherian species on the Paleocene side of the KPg boundary. This increase (e.g. from 11 extinct eutherian species in the Late Cretaceous to 139 in the early Tertiary) is viewed as supporting evidence for the *Explosive Model* (Archibald and Deutschman, 2001). Several studies have investigated if this apparent increase is an artifact related to limited sampling in the Late Cretaceous (Alroy, 1999; Benton et al., 2000; Archibald and Deutschman, 2001; Davies et al., 2017). The resulting quantitative analyses suggest that the explosive increase in morphological and taxonomic diversity after the KPg boundary is biologically significant and is not due to a poor fossil record in the Cretaceous (Alroy, 1999; Davies et al., 2017). Reconstructions of ancestral areas for placental mammals further suggest that the interordinal radiation of Boreoeutheria occurred in Eurasia and North America (Springer et al., 2011), areas that contain some of the best-known Late Cretaceous fossil localities. These results suggest that the current distribution of sampling localities should be sufficient to uncover Late Cretaceous crown boreoeutherian fossils if they are present (Phillips, 2016). A caveat is that there is no fossil record of Cretaceous eutherians in Africa so potential placental fossils on this continent remain unsampled (Phillips, 2016). Other landmasses with a poor or missing fossil record of eutherians from all or most of the Cretaceous include Antarctica, Madagascar, and India. Also, an important criticism of the *Explosive Model* of placental diversification is that it relies on the accurate phylogenetic placement of extinct eutherians from the Cretaceous as stem placentals. However, the placement of some extinct taxa is subject to significant uncertainty for a variety of reasons (see below). An additional criticism of extreme versions of the *Explosive Model* (e.g., O’Leary et al., 2013) is that the nucleotide substitution rates for basal branches of Placentalia would have been extremely high, more representative of DNA viruses than those typically observed in mammals, to fit the *Explosive Model* (Springer et al., 2013). While the *Explosive Model* is the hypothesis that is best supported by traditional interpretations of the fossil record, it has not yet been supported by any rigorous molecular analysis.

### Soft Explosive Model

The *Soft Explosive Model* allows for cladogenesis among the major superordinal groups (Xenarthra, Afrotheria, Laurasiatheria, and Euarchontoglires) in the Cretaceous, but places the remainder of placental interordinal diversification near or after the KPg boundary (Phillips, 2016) (**Figure 1B**). Like the *Explosive Model*, this hypothesis suggests that the rapid interordinal diversification seen after the KPg boundary occurred in response to ecospace filling in the absence of non-avian dinosaurs (Phillips, 2016; Phillips and Fruciano, 2018). The *Soft Explosive Model* does not preclude a few crown placentals from the late Cretaceous, but suggests that the vast majority of Late Cretaceous eutherians are stem placentals rather than members of Placentalia. However, as discussed below there are significant problems with the placement of extinct mammalian orders based on parsimony or likelihood analyses of morphological characters. Phillips (2016) suggested that Cretaceous dates for most interordinal splits, as are commonly recovered in studies that support the *Long Fuse Model*, are the result of rate transference errors that can be avoided by removing fossil calibrations for taxa that are large and/or long-lived. These taxa generally have slower rates of molecular evolution relative to small-bodied, short-lived mammals with shorter generation times that might better approximate most early placental taxa (Bromham, 2011). However, recent analyses have shown that not calibrating large or long-lived taxa can result in zombie lineages, where taxa have a fossil record that is older than the divergence time estimated from molecular data (Springer et al., 2017). While ghost lineages are the expected result of an incomplete fossil record (Springer and Lilje, 1988; Strauss and Sadler, 1989; Springer, 1990; Marshall, 1997), zombie lineages are logically impossible if extinct taxa have been correctly identified because divergence times cannot be younger than minimum ages implied by the fossil record (Springer et al., 2017). Indeed, omitting or using too few fossil calibrations for large or long-lived taxa biases analyses to underestimate the ages of these lineages, and can also drag the ages of deeper nodes towards the present (Springer et al., 2017). This debate has continued in the literature with the focus once more returning to the issue of fossil calibrations (Phillips and Fruciano, 2018), which due to their somewhat subjective nature are a long recognized and ongoing source of conflict in node-dating analyses (Yang and Rannala, 2006; Donoghue and Benton, 2007; Inoue et al., 2009; Pyron, 2009; Parham et al., 2012).

### Trans-KPg Model

The *Trans-KPg Model* is similar to the *Short Fuse Model* in suggesting that much of the interordinal diversification (cladogenesis) of placental mammals occurred after the KPg mass extinction. In contrast to the latter model, however, the *Trans-KPg Model* suggests that interordinal diversification was part of a continuous radiation in the Late Cretaceous and early Cenozoic that was uninterrupted by the KPg mass extinction (Liu et al., 2017a) (**Figure 1C**). The steady rate of interordinal diversification of placental mammals through time is proposed to coincide with a parallel radiation of herbivorous multituberculates in response to the gradual increase in ecological opportunity afforded by the rise of the angiosperms (Liu et al., 2017a). Similar to the *Soft Explosive Model*, timetrees that support the *Trans-KPg Model* are compromised by extensive zombie lineages (Gatesy and Springer, 2017; also see below), in addition to homology errors in the underlying data set (Gatesy and Springer, 2017). One reanalysis of Liu et al.’s (2017a) data set that purportedly corrected these homology errors yielded divergence time estimates that presumably contain the same host of zombie lineages as the first attempt because the authors claimed that the new divergence times were strongly correlated (0.9997) with the original divergence times (Liu et al., 2017b). A different reanalysis based on a revised suite of fossil calibrations supported the *Soft Explosive Model* (Phillips and Fruciano, 2018), further highlighting the sensitivity of timetrees to different node-based fossil calibration schemes.

### Long Fuse Model

The *Long Fuse Model* posits that all or most interordinal cladogenesis occurred in the Cretaceous whereas the majority of intraordinal diversification took place after the KPg boundary (Archibald and Deutschman, 2001) (**Figure 1D**). Under this scenario, the initial diversification of placental mammals began in the Cretaceous, possibly in response to the Cretaceous Terrestrial Revolution and the associated diversification of flowering plants and insects (Meredith et al., 2011). Like the *Explosive Model*, the *Long Fuse Model* suggests an important role for the KPg boundary event, but restricts its impact to intraordinal splitting and ecological/phenotypic diversification, which exploded after the KPg mass extinction event in response to newly available niche space (Meredith et al., 2011). This hypothesis is most strongly favored by analyses of molecular datasets comprising multiple gene fragments for small and large numbers of taxa (Kumar and Hedges, 1998; Eizirik et al., 2001; Murphy et al., 2001a; Murphy et al., 2001b; Springer et al., 2003; Springer et al., 2005; Murphy et al., 2007; Meredith et al., 2011; Lartillot and Delsuc, 2012; Emerling et al., 2015; Hedges et al., 2015; Foley et al., 2016; Springer et al., 2017) and genome wide data (Wildman et al., 2007; dos Reis et al., 2012; dos Reis et al., 2014; Tarver et al., 2016; Wu et al., 2017).

The *Long Fuse Model* predicts the occurrence of placental fossils deep in the Cretaceous. Possible eutherian forms are recognized in the fossil record as far back as the Jurassic with the discovery of *Juramaia sinensis* in China (Luo et al., 2011), although the phylogenetic placement of this taxon is contentious and some analyses have recovered *Juramaia* as a stem therian (e.g., Krause et al., 2014). Indeed, relationships among various eutherian forms that appear in the fossil record prior to the KPg boundary are controversial, with much debate centering over the correct assignment of extinct taxa to the stem of Placentalia or to the crown clade. This problem is exacerbated by the fragmentary skeletal remains that have been recovered for many of these taxa.

Fossils attributed to the Late Cretaceous families Zalambdalestidae and Zhelestidae were originally considered placentals (Archibald, 1996; Archibald et al., 2001). Specifically, cladistic analyses suggested that zalambdalestids represent a paraphyletic stem group to Glires (lagomorphs and rodents) whereas zhelestids form a clade with Ungulata (Archibald et al., 2001). Subsequent analyses with expanded taxon sampling have excluded zalambdalestids and zhelestids from crown Placentalia, instead recovering these fossils as stem placentals (Wible et al., 2009; Archibald and Averianov, 2012; O’Leary et al., 2013; Zhou et al., 2013). These contrasting results also highlight the importance of missing data. Another candidate crown placental from the Cretaceous is *Protungulatum coombsi*, which is known from at least 300,000 years before the KPg boundary in the Late Cretaceous Hell Creek Formation of Montana (Archibald et al., 2011). O’Leary et al. (2013) analyses of the morphological data set for mammals with the largest number (4541) of characters, as well as a combined analyses of this matrix with DNA data, reconstructed the position of *Protungulatum* as a crown laurasiatherian, thereby providing some paleontological support for a Cretaceous origin of Placentalia. However, like many fossil eutherians the position of *Protungulatum* is controversial. More recently, (Halliday et al. 2017; 2019) recovered a stem placental position for *Protungulatum*. Another intriguing candidate for membership in Placentalia is *Gypsonictops*, which has now been reported from the Turonian (93.9–89.8 Ma) (Cohen and Cifelli, 2015; Cohen, 2017; Halliday et al., 2019). Halliday et al. (2019) recovered *Gypsonictops* (family Gypsonictopidae) and *Leptictis* (family Leptictidae) as sister taxa just outside of Placentalia. Numerous authors have also recognized an association of these families together in Leptictida (Gunnell et al., 2007; Wible et al., 2007; Wible et al., 2009). O’Leary et al. (2013) included *Leptictis* in their cladistic analysis of 4541 characters and recovered this taxon inside of Placentalia. However, Leptictidae is only known from the Cenozoic and its inclusion in Placentalia does not mandate a Cretaceous age for Placentalia. Still, taken together, the results of O’Leary et al. (2013) and Halliday et al. (2019) hint at the possible inclusion of Leptictida in crown Placentalia. More specifically, if Leptictidae and Gypsonictopidae are sister taxa, and if this clade is positioned in crown Placentalia rather than the stem group, then the main paleontological objection to the *Long Fuse Model* would be largely blunted.

### Short Fuse Model

The *Short Fuse Model* posits interordinal and some intraordinal diversification of placental mammals well back in the Late (Upper) Cretaceous (Archibald and Deutschman, 2001) (**Figure 1E**). The initiation of interordinal cladogenesis may even extend as far back as the Upper Jurassic (Archibald and Deutschman, 2001). According to this model, the mass extinction event at the KPg boundary did not play a significant role in the interordinal diversification of present-day mammals nor the ecomorphological divergence of many ordinal level crown clades. Unlike the *Explosive* and *Long Fuse Models*, both of which are widely advocated in the literature, support for the *Short Fuse Model* is restricted to a relatively small number of studies. These include early molecular clock analyses (e.g., Kumar and Hedges, 1998), a supertree analysis (Bininda-Emonds et al., 2007), and more recently morphological clock studies (Puttick et al., 2016; Caldas and Schrago, 2019). The most explicit support for the *Short Fuse Model* comes from Bininda-Emonds et al. (2007), who used a matrix representation with parsimony approach to build a supertree representing ∼99% of mammalian species-level diversity. However, the molecular dating analysis employed local molecular clocks and a pure birth model to interpolate some divergence times. Bininda-Emonds et al. (2007) concluded that the KPg extinction had no effect on the diversification of extant lineages, and instead suggested that increased diversification in the Eocene may have been triggered by the Early Eocene Climatic Optimum (Bininda-Emonds et al., 2007). The conclusion that extant lineages experienced accelerated rates of diversification in the Eocene was not supported by a subsequent study that employed relaxed clock methods (Meredith et al., 2011). Puttick et al. (2016) performed tip dating with the morphological data set (4,541 characters) of O’Leary et al. (2013) and recovered interordinal and intraordinal divergence times for the placental radiation that are even older than those of Bininda-Emonds et al. (2007) (see *Challenges for Tip Dating*).

By contrast with these timetree studies, Tavaré et al. (2002) and Wilkinson et al. (2011) used modeling approaches to address the question of whether or not divergence times within crown Primates (Euprimates) extend as far back as the KPg boundary. If intraordinal divergence times in Primates extend into the Mesozoic, then interordinal divergences for deeper nodes must be at least this old. These modeling approaches incorporated parameters for fossil preservation rates, the mean longevity of fossil primate species, and the number of extant primate species. Tavaré et al. (2002) concluded that crown Primates last shared a common ancestor ∼81.5 Ma. Wilkinson et al. (2011) obtained posterior estimates of divergence times for several nodes within Primates based on their modeling approach and then used these estimates as priors in an MCMC analysis with DNA sequences. Similar to Tavaré et al. (2002); Wilkinson et al. (2011) concluded that Primates last shared a common ancestor ∼84.5 Ma. Thus, both of these studies are consistent with the predictions of the *Short Fuse Model*. However, Phillips (2016) criticized several assumptions of these models including logistic species accumulation and long times to speciation (2–3 myr), both of which favor a long period of missing history early in primate evolution.

## Node Dating and Beyond

Several problems are potentially of concern for node- tip-, and fossilized birth–death dating methods that can be applied to the placental radiation. Other shortcomings are restricted to a subset of these methods. In this section we first address common problems and then examine unique problems that are associated with specific methods.

### Homology

**Figure 2:**
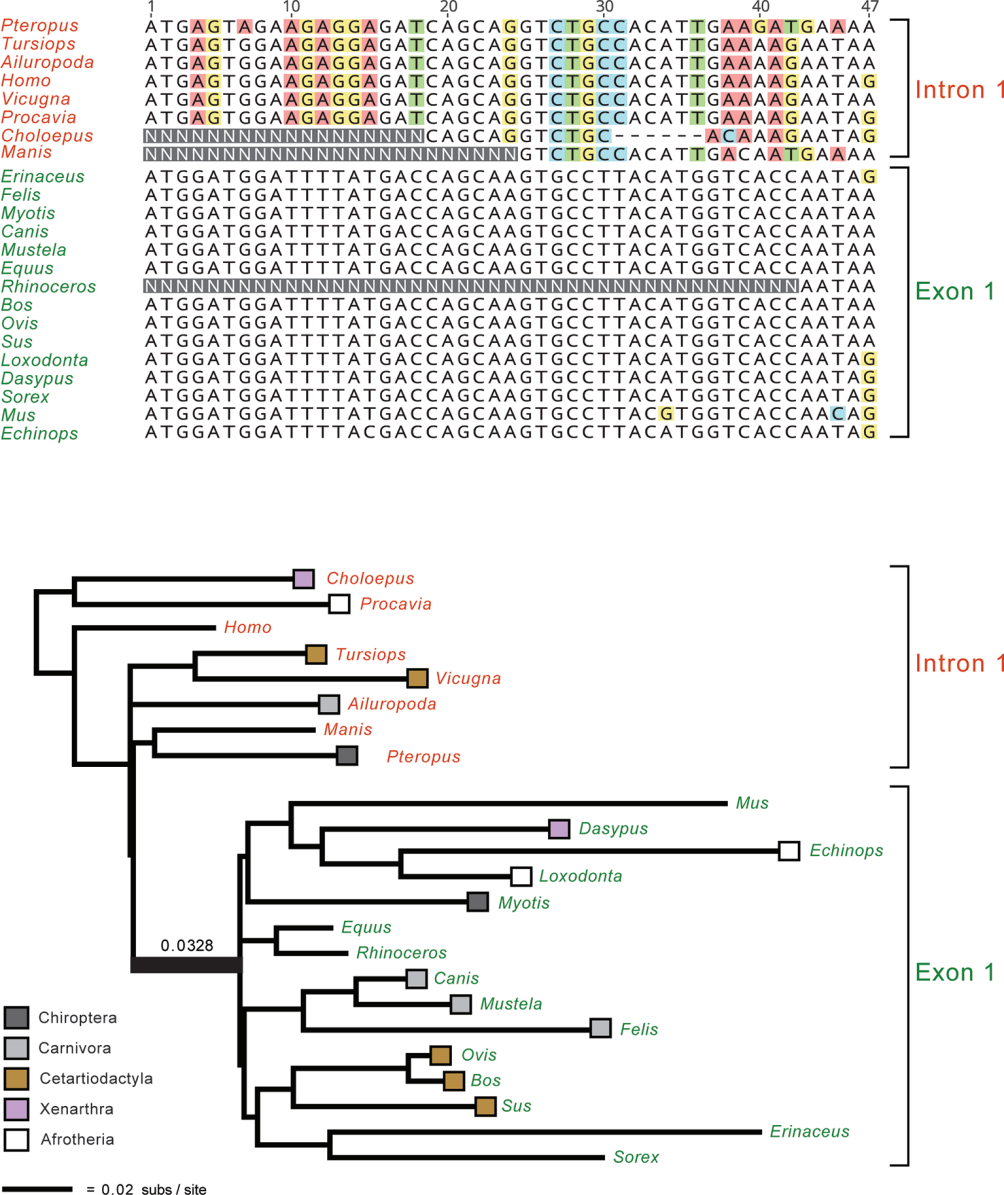
Example of a homology problem from Chen et al.’s (2017) phylogenomic data set for Laurasiatheria and outgroups. Partial *ETV1* gene alignment (top) and gene tree for the full *ETV1* alignment are shown. Protein-coding sequences for 15 taxa (green lettering) are for exon 1 and begin on the start codon ATG, but the first eight taxa in the alignment (red lettering) instead are represented by sequence from intron 1 of *ETV1*. Faulty annotation and subsequent misalignment of protein-coding sequence to non-coding sequence results in 20 ‘pseudo-synapomorphies’ for a clade that contradicts five well-established mammalian clades. The long internal branch that subtends this clade, 0.0328 substitutions per site, is indicated. Nucleotides that differ from the majority nucleotide at each position in the alignment are highlighted in colored boxes.

An important issue for all molecular timetree methods is the underlying quality of the DNA or protein alignments. In the Sanger sequencing era, it was straightforward to inspect individual alignments for misaligned regions or problematic sequences from smaller sets of orthologous genes. Similarly, gene trees were routinely inspected for red flags such as unexpected relationships that may indicate contamination or paralogy. However, it is no longer practical to inspect/edit thousands of alignments that are tens or even hundreds of kilobases in length and contain hundreds of taxa. Nevertheless, this does not excuse researchers from assessing the quality of their alignments and gene trees. Indeed, numerous phylogenomic data sets (Struck et al., 2011; Chiari et al., 2012; Song et al., 2012; Kumar et al., 2013; Jarvis et al., 2014; Feijoo and Parada, 2017; Chen et al., 2017; Liu et al., 2017a) contain alignments with homology problems that impact the results and main conclusions of these studies (Struck, 2013; Springer and Gatesy, 2016; Brown and Thomson, 2017; Gatesy and Springer, 2017; Springer and Gatesy, 2018a; Springer and Gatesy, 2018b). These problems could have been avoided with appropriate screening procedures to flag problematic alignments and gene trees. **Figure 2** shows an example of yet another phylogenomic data set (Chen et al., 2017) with large-scale homology problems that impact the major conclusions of this study. Even without inspecting all of the constituent alignments and gene trees, it is possible to ascertain if there are systematic problems *via* targeted or even random sampling of the individual alignments and trees. One approach for targeted inspection is to view alignments that correspond to the gene trees with the highest Robinson-Foulds (RF) distances (Robinson and Foulds, 1981). RF Distances Filter (Simmons et al., 2016) is especially useful for this purpose and outputs normalized RF distances between gene trees (or between gene trees and a species tree) that range from 0 for identical trees to 1 for trees with no internal branches in common. Problematic sequence alignments as shown in **Figure 2** are not difficult to recognize, especially for a trained systematist who is acquainted with the taxonomy of their group. Reciprocal BLAST searches and re-alignments, sometimes in conjunction with new phylogenetic analyses, can be used to verify if problematic regions of an alignment correspond to orthologous regions of the same gene or not (Springer and Gatesy, 2018a; Springer and Gatesy, 2018b). Similarly, a targeted approach may be used to inspect all alignments with long branches that exceed a specified threshold (Mason et al., 2016). Springer and Gatesy (2018b) used both of these approaches (highest RF distances, long branches) to identify alignments with orthology problems for several phylogenomic data sets including Kumar et al.’s (2013) data set for Euarchontoglires and Jarvis et al.’s (2014) data set for birds. We agree with Bromham (2019, p. 3) that the "safest approach is to only analyze those alignments for which you are certain of homology for all columns and rows, resisting the temptation to analyze unverified alignments for the sake of expedience." Homology errors in alignments will be propagated in all subsequent steps (e.g., phylogeny reconstruction, estimation of divergence dates) and should be avoided. Ongoing efforts to develop new methods to screen genomic alignments (e.g., Ali et al., 2019) for such errors should reduce this source of error moving forward. It is also important for authors to make all gene alignments available so that *ad hoc* criteria used to exclude genes (or regions thereof) can be evaluated by other researchers.

### Zombie and Ghost Lineages

**Figure 3:**
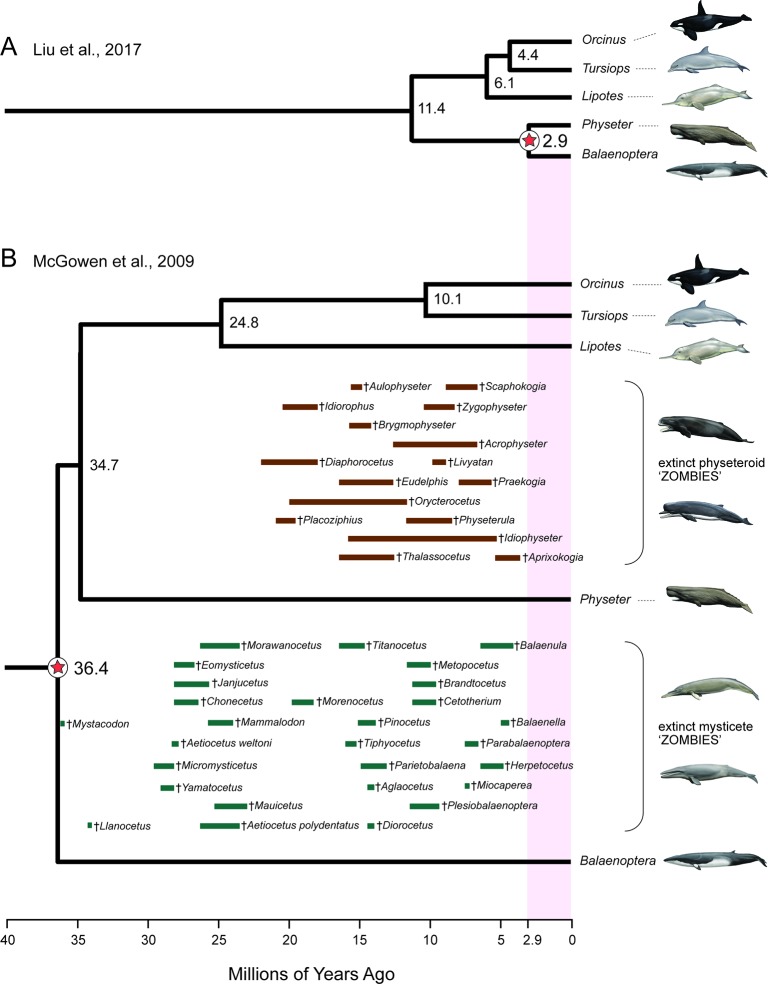
Example of ‘zombie’ whale lineages implied by the timetree for mammals of Liu et al. (2017a). Due to inadequate density of fossil calibrations in this molecular clock study, the slowly evolving cetacean clade shows extremely shallow divergences **(A)** relative to previous molecular clock analyses such as McGowen et al. (2009) **(B)**. Numerous extinct sperm whales (Physeteroidea) and baleen whales (Mysticeti) are found in strata that are much older than the divergence time estimate between *Physeter* (giant sperm whale) and *Balaenoptera* (rorqual baleen whale) in **(A)** but not in**(B)**. Geological range estimates for extinct mysticetes (green bars) and physeteroids (brown bars) are from Marx and Fordyce (2015) and Lambert et al. (2017a; 2017b). Paintings are by C. Buell.

An additional red flag for timetree analyses is the occurrence of zombie lineages, where estimated divergence times are younger than minimum ages implied by fossils (Springer and Gatesy, 2016; Springer and Gatesy, 2018c). Zombie lineages are evident in several recent studies that have addressed the timing of the placental radiation (Phillips, 2016; Sato et al., 2016; Liu et al., 2017a). The most extreme example is Liu et al. (2017a) where the estimated divergence date for sperm whale [a toothed whale (Odontoceti)] to minke whale [a baleen whale (Mysticeti)] is only 2.9 Ma. This estimated date is more than an order of magnitude younger than the age of the oldest mysticete fossil (*Mystacodon*, 36.4 Ma) (Gatesy and Springer, 2017; Lambert et al., 2017a; de Muizon et al., 2019) and is also younger than numerous extinct mysticete and physeteroid (sperm whale) genera (**Figure 3**). By contrast, McGowen et al.’s (2009) timetree for Cetacea accommodates all of these fossils without any zombie lineages (**Figure 3**). At the opposite end of the spectrum, Barido-Sottani et al.’s (2019) fossilized birth–death analysis of Cetacea resulted in excessively long ghost lineages when fossil ages were estimated from an uncertain age range using midpoints or randomly sampled from these same age ranges. Specifically, the most recent common ancestor of crown Cetacea was estimated at > 60 Ma with midpoint ages and > 50 Ma with random draws from uncertain age ranges. The former date is more than 23 million years older than the earliest known crown cetaceans (Lambert et al., 2017a; de Muizon et al., 2019) and ten million years older than *Ambulocetus* (= walking whale), which is an early transitional form (stem Cetacea) that retained short limbs and large feet for swimming (Thewissen et al., 1996; Madar et al., 2002). Zombie lineages and ghost lineages should both be carefully compared to the fossil record in timetree analyses. Long ghost lineages are sometimes required because of a poor fossil record, as is the case for craseonycterid and myzopodid bats (Teeling et al., 2005), but the long ghost lineages for Cetacea implied by Barido-Sottani et al.’s (2019) analyses are less reasonable given the much more complete fossil record for cetaceans than for craseonycterid or myzopodid bats.

### Phylogenetic Placement of Fossils

**Figure 4:**
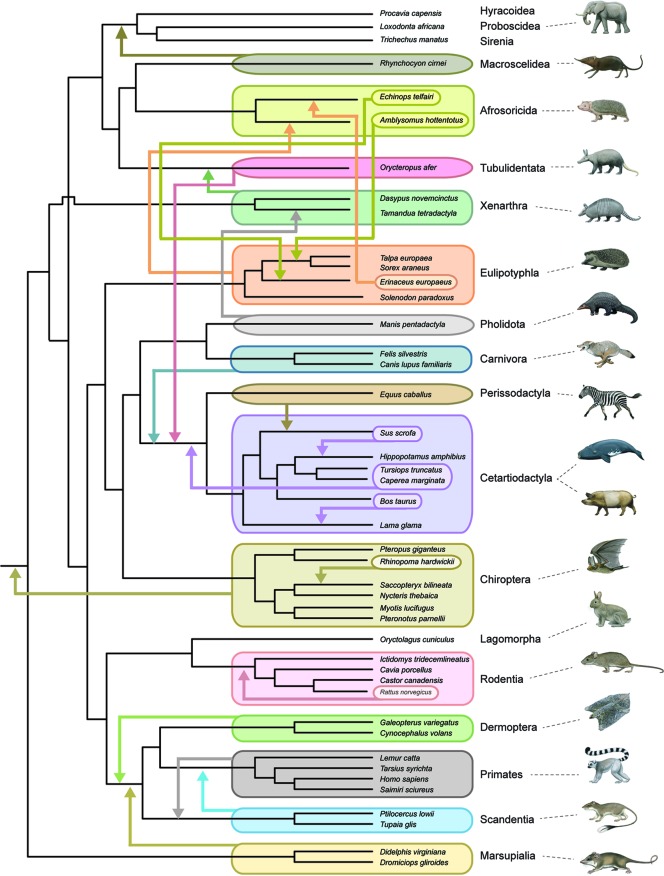
Summary of pseudoextinction results for the reanalysis of morphological data from Morphobank Project 773 (O’Leary et al., 2013). Analyses were performed with a molecular scaffold that was based on robustly supported clades (>95% bootstrap support) from Meredith et al.’s (2011) phylogenetic analysis of 26 nuclear loci. The molecular scaffold included several polytomies that are not yet confidently resolved by molecular data: trichotomy at root of Placentalia (Afrotheria, Boreoeutheria, Xenarthra), paenungulate trichotomy (Hyracoidea, Proboscidea, Sirenia), Euarchontoglires trichotomy (Primatomorpha, Glires, Scandentia), and Laurasiatheria polytomy (Carnivora+Pholidota, Chiroptera, Cetartiodactyla, Perissodactyla). Pseudoextinct taxa were made pseudoextinct by recoding soft tissue characters as missing and deleting the pseudoextinct taxon from the molecular scaffold. Maximum parsimony analyses of 19 ordinal level taxa were individually executed with PAUP 4.0a165 (Swofford, 2002) and compared to the master scaffold. Parsimony analyses for each pseudoextinct clade were performed with 1000 random input orders of taxa and tree-bisection and reconnection branch swapping. Mammalian orders that showed shifts in phylogenetic position in these analyses are indicated by arrows that show the movements of entire clades as well as the repositioning of subtaxa within or among orders. Only four orders (Lagomorpha, Hyracoidea, Proboscidea, Sirenia) did not show changes to phylogenetic relationships in these analyses. Monotreme outgroups were included in the original analysis but were pruned from the tree shown here. Paintings are by C. Buell.

All timetree methods are critically dependent on the accurate phylogenetic placement of extinct taxa, whether through *a priori* decisions based on previous analyses and observations (node-dating, fossilized birth–death dating) or through the simultaneous estimation of phylogenetic relationships and divergence times (tip dating). This task is especially difficult for placental orders because of widespread ecomorphological convergence and correlated character evolution (Springer et al., 2007; Springer et al., 2013; Springer et al., 2017). For example, highly specialized myrmecophagy has evolved independently in Old World pangolins (Pholidota), African aardvarks (Tubulidentata), and New World anteaters (Xenarthra). Some or all of these taxa routinely cluster together in morphological cladistic analyses (Novacek, 1986; O’Leary et al., 2013). Darwin (1859) was aware of the general problem of ecomorphological convergence and noted that adaptation to similar conditions will conceal, rather than reveal, genealogical relationships. Total evidence phylogenetic analyses that combine morphological and molecular data matrices together can mitigate this problem for extant taxa, but there is no guarantee that extinct taxa will be accurately placed based on morphological data alone, especially if extinct taxa are from orders (e.g., Creodonta, Mesonychia) that are only distantly related to living forms. One approach to assess the severity of this problem is through pseudoextinction analyses that render all representatives of a living order extinct by retaining osteological characters but recoding molecular and soft morphological characters as missing. The logic behind this approach is that only hard parts are typically fossilized in extinct taxa (Springer et al., 2007). Springer et al. (2007) showed that the majority of placental orders moved to different phylogenetic positions when they were treated as pseudoextinct and also that some of these orders became polyphyletic. One caveat is that Springer et al. (2007) examined a relatively small osteological data set of 185 characters from Asher et al. (2003) and raised the possibility that larger morphological data sets would overcome the problems that beset smaller data sets if these problems were statistical in nature (e.g., see Sterli et al., 2013) and resulted from small sample size. O’Leary et al.’s (2013) massive morphological data set (4,541 phenomic characters) provided an opportunity to re-evaluate the effects of pseudoextinction without the potential problem of small sample size. **Figure 4** shows the results of a pseudoextinction analysis with maximum parsimony for the extant orders of placental mammals and marsupial outgroups. As was the case in Springer et al.’s (2007; 2008) pseudoextinction analyses, the majority of placental orders moved to a different interordinal location when pseudoextinct (i.e., treated as fossils and just coded for hard parts). In addition, three of these orders (Afrosoricida, Cetartiodactyla, Eulipotyphla) become para- or polyphyletic (**Figure 4**). Distantly related insectivores (Afrosoricida, Eulipotyphla) group with each other, and all three orders with highly specialized myrmecophages (Xenarthra, Pholidota, Tubulidentata) cluster with one of the other myrmecophagous orders when treated as pseudoextinct. These results suggest that an entirely extinct clade of myrmecophagous placental mammals might join with one of the other myrmecophagous groups even if the true phylogenetic position of this extinct group is elsewhere in the overall tree. For example, the phylogenetic position of *Eurotamandua*, an enigmatic myrmecophage from the middle Eocene of Europe, is likely to be conflated with other myrmecophages such as pangolins or anteaters even if myrmecophagy originated independently in this taxon. Indeed, previous assessments of the phylogenetic affinities of this taxon based on putative synapomorphies and cladistic analyses suggest that *Eurotamandua* is closely related to Vermilingua (anteaters) (Storch, 1981), to Pholidota (pangolins) (McKenna and Bell, 1997), to Palaenodonta (an extinct relative of Pholidota) (Rose, 1999), or to Xenarthra (Halliday et al., 2019). There are also cases of extinct taxa whose phylogenetic position shifts to a seemingly less accurate position when morphological data for all taxa (extinct and extant) are analyzed in combination with molecular data for extant taxa in a total evidence analyses. One example is the extinct taxon *Rodhocetus*, which belongs to the stem cetacean family Rodhocetidae. The position of this taxon based on morphology only is with other cetaceans (Gatesy et al., 2013; O’Leary et al., 2013). However, *Rodhocetus* is outside of a clade that contains other cetartiodactyls plus perissodactyls in O’Leary et al. (2013) total evidence analysis. This result shows that molecular data do not always improve the phylogenetic placement of extinct taxa, especially for incompletely preserved fossils. *Rodhocetus* is only scored for 386 of 4,541 characters in O’Leary et al.’s phenomic character matrix.

The inclusion of extinct and extant taxa in the same analysis has the potential to break up long branches and improve phylogenetic accuracy, but diachronous terminals (i.e., terminals of different ages) may also create problems for morphological cladistic analysis. Namely, diachronous terminals create opportunities for long-branch misplacement because root to tip distances are longer for extant taxa than for fossils (Wang et al., 2005; Springer et al., 2017). This problem mimics lineage-specific rate variation in analyses of molecular data with extant taxa. The phylogenetic placement of fossils can also be negatively impacted by the inevitable bias of the fossil record to preserve hard (biomineralized) morphological structures. This bias can systematically distort phylogeny. Specifically, Sansom and Wills (2013) showed that fossils are more likely to move stemward than crownward when they are only known for biomineralized characters. The causes of stemward slippage are not entirely clear, although Sansom and Wills (2013) suggest that fundamental taphonomic biases associated with the preservation of hard versus soft part characters cause fossils to be interpreted as erroneously primitive. The result of this "stemward slippage" is that divergence dates will be underestimated (Sansom and Wills, 2013). Finally, a recent study on morphological evolution in placental mammals concluded that it may be very difficult to distinguish early members of the major placental groups from stem eutherians on the basis of skeletal and dental characters because Cretaceous forms were not ecologically diverse and may appear very similar to each other (Halliday et al., 2019). In a similar vein, previous authors hypothesized that placentals from the Cretaceous were small and may have diversified phylogenetically before they diverged morphologically and acquired the diagnostic features of crown placental orders (Easteal, 1999; Madsen et al., 2001; Springer and Murphy, 2007). For these reasons, it is difficult to have confidence in the phylogenetic placement of fossils that are only distantly related to extant forms. In addition, these problems are more likely to impact deeper nodes because the placement of extinct taxa becomes more uncertain with increasing phylogenetic depth.

In spite of potential difficulties with convergent evolution and diachronous terminals, fossils remain fundamentally important for understanding the timing of the placental radiation. Similarly, fossils are critical for deciphering sequences of character evolution because they record unique combinations of morphological characters that are unknown in living mammals (Lee and Palci, 2015). On the other hand, the misplacement of these fossils in a phylogenetic analysis may distort the resulting estimates of both divergence times and ancestral character states. To the extent that we are confident in the phylogenetic placement of fossils we may also be more confident in both timetree analyses and ancestral character state transformations. Here, the placement of fossils may be more reliable when they belong to groups that also have extensive living representatives such as Tenrecidae (Asher and Hofreiter, 2006), Primates (Pattinson et al., 2015), and Rodentia (Asher et al., 2019). However, the placement of extinct taxa without living representatives remains more elusive. For example, the entirely extinct Plesiadapiformes are generally recognized as a paraphyletic taxon at the base of Euprimates (Silcox et al., 2017), but some cladistic analyses with more comprehensive taxon sampling place the earliest known plesiadapiform genus, *Purgatorius*, outside of Placentalia (Halliday et al., 2017; Halliday et al., 2019). Similarly, Gheerbrant et al. (2018) recovered the extinct order Embrithopoda as a clade of stem tethytheres, but other analyses have positioned this order elsewhere within Paenungulata or even deeper in Afrotheria (Tabuce et al., 2007; Cooper et al., 2014; Erdal et al., 2016). An even more difficult fossil group is Anagalida, which minimally includes the families Anagalidae and Pseudictopidae. Representatives of these families have been recovered as stem Glires, the sister taxon to Macroscelidea, or even as stem placentals in different phylogenetic analyses (Meng et al., 2003; Meng, 2004; Asher et al., 2019).

There are no easy solutions for elucidating ecomorphological convergence among extant and extinct placental mammals. One positive result for a longstanding phylogenetic problem concerns the phylogenetic placement of two recently extinct orders of South American ungulates, Notoungulata and Litopterna. Morphological studies have placed one or both of these orders in a variety of different locations on the placental tree. O’Leary et al. (2013) included a representative of each of these orders in their phylogenetic analysis of the mammalian radiation. They recovered a stem euungulate (Cetartiodactyla + Perissodactyla) position for *Protolipterna*, an early representative of the order Litopterna, and a nested position within Paenungulata (Proboscidea + Sirenia + Hyracoidea) for *Thomashuxleya*, a representative of the order Notoungulata. More recently, amino acid sequences for ancient collagen molecules from extinct members of these orders have been determined using mass spectrometry (Buckley, 2015; Welker et al., 2015). Phylogenetic analyses based on these sequences show that the representative litoptern (*Macrauchenia*) and notoungulate (*Toxodon*) are sister taxa to each other and that this monophyletic group is the sister taxon to Perissodactyla (Buckley, 2015; Welker et al., 2015). This clade was named Panperissodactyla (Welker et al., 2015) and was partially corroborated by a phylogenetic analysis of mitogenomic sequences by Westbury et al. (2017) that demonstrated a sister-group relationship between Litopterna (*Macrauchenia*) and Perissodactyla (Notoungulata not included in the analysis). Molecular sequences are not immune to homoplasy, as for example the lysozyme protein in foregut fermenting ruminants, colobus monkeys, and the hoatzin (Kornegay et al., 1994) and a handful of hearing proteins such as prestin in echolocating bats and toothed whales (Liu et al., 2010; Davies et al., 2012). However, convergent changes in these genes are limited to replacement substitutions and do not extend broadly across the genome to other loci. Liu et al. (2010) found that echolocating dolphins cluster with echolocating horseshoe and Old World leaf-nosed bats based on amino acid sequences for prestin, but analyses based on nucleotide alignments, which index both replacement and silent substitutions, recovered the accepted species tree and were not misled by convergence. Further, we are unaware of any phylogenomic analyses that group ruminants with colobus monkeys or echolocating bats with toothed whales. By contrast, there are several groups of ecomorphologically similar mammals (e.g., ant and termite eaters) that group together based on O’Leary et al. (2013) massive data set that includes morphological characters from many different parts of the body (Springer et al., 2013). Finally, given that Panperissodactyla is supported by independently segregating molecular markers (mitogenomes and collagen protein sequences), it seems unlikely that this relationship is driven by convergent evolution.

### Challenges for Node Dating

Since 2003, node dating with a relaxed molecular clock has been the main approach used to estimate divergence times in different taxa including the timing of the placental radiation. Node dating is based on calibrating internal nodes against the fossil record (Ronquist et al., 2012). It is easy to apply with limited information from the fossil record, but like other methods (i.e., tip dating, fossilized birth-death dating) is not guaranteed to yield accurate divergence dates given some of the problems noted below. Node dating is implemented in several popular programs (e.g., mcmctree, BEAST). This approach does not require a morphological data matrix and can be implemented with both soft and/or hard-bounded calibrations. One potential problem with node dating is the use of unrelated priors (treewide prior, node-specific calibration) for each calibrated node (Heath et al., 2014). However, this problem can be avoided by applying a birth–death process to the uncalibrated nodes conditioned on the calibrated nodes (Yang and Rannala, 2006). A more serious problem is that probability densities for maximum age bounds are usually based on subjective or arbitrary criteria and are rarely informed by biological processes and/or detailed knowledge of the fossil record (Benton and Donoghue, 2007; Ho and Phillips, 2009; Heath et al., 2014; Arcila et al., 2015; Lee and Palci, 2015). The fossilization process is modeled only indirectly in node dating and in isolation from other forms of data (Heath et al., 2014). Models for branch-rate variation (e.g., lognormal, exponential) and its deployment (e.g., independent, autocorrelated) are drawn from statistical distributions that are convenient and tractable, but not necessarily reflective of real biological processes. This same criticism applies to tip dating methods (below). Meredith et al. (2011) showed that autocorrelated and independent models for the deployment of rate variation both perform poorly unless there is a dense network of calibrated nodes to combat (1) zombie lineages in large-bodied mammals with slow rates of evolution, and (2) excessively old divergences in small-bodied mammals with fast rates of evolution. Trends toward increased body size in extant mammalian orders may bias estimates of interordinal divergence times if calibrations are applied to large-bodied clades (Phillips, 2016), but this problem can be partially mitigated with hard-bounded constraints that enforce maximum ages (Meredith et al., 2011) and/or the exclusion of large-bodied taxa from timetree analyses of placental mammals (Springer et al., 2003; Springer et al., 2017).

### Challenges for Tip Dating

In tip dating, morphological characters are coded for extinct and extant taxa and included in a combined data matrix that also includes molecular data for extant taxa (and in some cases recently extinct taxa). Tip dating employs a single probabilistic model that encompasses all of the different data types (fossil ages, molecular data matrix, and morphological data matrix) and then jointly estimates all of the model parameters, including a dated phylogeny, in a single analysis. However, current implementations of tip dating have limitations. First, the phylogenetic placement of extinct taxa based on morphological data may be highly inaccurate because of correlated homoplasy, which occurs when multiple characters are correlated with each other and with the same environmental variables (Springer et al., 2007; Springer et al., 2017). Such correlations may be driven by adaptation to similar niches or by developmental constraints. The inclusion of molecular data can help to tease apart homology from homoplasy for extant taxa, but most fossils can only be scored for morphological data with their attendant problems of correlated character evolution. Second, the delineation of morphological characters and character states is intrinsically more subjective than is the case for molecular data, where there are just four nucleotides for DNA and 20 amino acids for proteins. Third, the notion of morphological clocks is problematic. Puttick et al. (2016) analyzed O’Leary et al. (2013) phenomic character matrix for extinct and extant mammals with a morphological clock model and obtained divergence time estimates for the most recent common ancestor of Placentalia that range from Late Jurassic (146.2 Ma) to Early Cretaceous (132.2 Ma) in age, much older than node-dating estimates based on molecular data sets that are generally in the range of 100–90 million years (Meredith et al., 2011; dos Reis et al., 2012; Emerling et al., 2015; Foley et al., 2016; Tarver et al., 2016; Springer et al., 2017). Similarly, Puttick et al. (2016) estimated dates for the most recent common ancestors of other superordinal groups that are consistently older than dates based on relaxed molecular clocks. Afrotheria (138.5–123.6 Ma), Euarchontoglires (139.1–125.1 Ma), and Laurasiatheria (142.6–128.3 Ma) all have dates that are tens of millions of years older than relaxed clock studies. Puttick et al.’s (2016) analyses also recovered Cretaceous dates for several crown orders including Cetartiodactyla (98.8–85.6 Ma), Chiroptera (88–80 Ma), and Eulipotyphla (106–91.2 Ma). Puttick et al. (2016) concluded that current implementations of tip dating analyses are prone to estimate ancient divergence estimates when based solely on morphological data. These authors recommended that the results of such analyses be treated with caution. Caldas and Schrago (2019) compared the results of molecular and morphological clocks with internal node calibrations and found that the majority of estimated ages were older with the morphological clock than the molecular clock. However, Caldas and Schrago (2019) estimated interordinal ages based on the morphological clock are younger than Puttick et al. (2016) estimated ages based on the morphological clock with tip dating. Taken together, the results of these studies (Puttick et al., 2016; Caldas and Schrago, 2019) suggest that morphological clocks and tip dating both contribute to older ages than are typically recovered with molecular clocks and node dating for placental mammals.

Models for morphological character evolution, such as the Mk model (Lewis, 2001), have been borrowed from molecular evolution as if morphological characters evolve under the same model as molecular characters. Molecular models may be tractable, but are unlikely to reflect realistic morphological character evolution. For example, most molecular models assume uniform branch rates, so that the probabilities of change for all characters, whether fast or slow, increase or decrease in concert with each other on each branch (Goloboff et al., 2018). As discussed by Goloboff et al. (2018), this assumption seems especially implausible for morphology. Finally, the collection of morphological data matrices is time consuming and expensive relative to the amount of data returned, and is not practical for most taxa on the scale of O’Leary et al.’s (2013) data set with > 4,500 phenomic characters for 86 mammaliaform taxa. Nevertheless, the development of these data matrices is crucial for various aspects of timetree estimation, either indirectly for node dating approaches or directly for tip dating approaches.

### Challenges for Fossilized Birth–Death Dating

The fossilized birth–death model serves as a single prior probability distribution for divergence time dating that is used to calibrate and estimate node ages. Arbitrary calibration densities are not required as is the case for node dating. Indeed, the only assumptions are: (i) constant speciation rate, (ii) constant extinction rate, (iii) fossils are recovered along branches of the species tree according to a Poisson process, and (iv) each extant species is sampled with probability *p*. The original implementation of fossilized birth–death dating is the DPPDiv program of Heath et al. (2014). One limitation of this version of fossilized birth–death dating is that it does not allow for the inclusion of morphological characters in the analysis and only considers the age of each fossil. DPPDiv therefore requires the assignment of fossils to specific calibration nodes in the phylogeny based on prior information as is also true for node dating. More recently, fossilized birth–death dating has been combined with tip dating in BEAST2 (Gavryushkina et al., 2017), but this requires molecular and/or morphological data matrices for fossil and extant taxa and is not currently an option for most mammalian clades. In addition, fossilized birth–death dating assumes constant speciation and extinction rates and may be ill suited to investigate the timing of the placental radiation that spans the KPg mass extinction in four of five evolutionary models (**Figure 1**). Constant speciation and extinction are also unlikely to hold across diverse taxa with widely varying life histories. For example, speciation rates in the order Rodentia (> 2,000 extant species) have historically been much higher than in the order Tubulidentata (one extant species). We expect that future versions of fossilized birth–death dating may allow for different speciation and extinction rates in different sectors of a phylogenetic tree. Third, it is unlikely that fossils are recovered along branches according to a Poisson process. Rather, fossil recovery rates are spatially and temporally non-uniform and vary across different continents, time periods, taxonomic groups, and depositional environments (Holland, 2016). An additional issue for fossilized birth–death dating pertains to the sampling of fossils with imprecise ages that are represented as age ranges in the literature or in the Paleobiology Database.

Heath et al. (2014) original description of the fossilized birth–death method for timetree estimation provided an illustration of their approach with an empirical data set for Ursidae (bears). In this example, Heath et al. (2014) employed a molecular data set that included complete mitogenomes and a single nuclear gene, and randomly sampled each extinct ursid and fossil outgroup with an imprecise age from a uniform distribution of its given range. This procedure resulted in divergence dates for Ursidae that are similar to the mitogenomic node-based estimates of Krause et al. (2008). These results suggest that fossilized birth–death dating can recover dates that are generally in line with dates that are estimated with other accepted methods. At the same time, Heath et al. (2014) suggested that it would be preferable to treat fossil ages as random variables by placing prior densities on fossil occurrence times conditional on their estimated age ranges. More recently, Barido-Sottani et al. (2019) found that fixing fossil ages to the midpoint or a random point drawn from within the stratigraphic age range resulted in biased estimates of divergence times. Specifically, estimated ages for Cetacea were much older than other studies (e.g., Steeman et al., 2009; McGowen et al., 2009; Marx and Fordyce, 2015) and imply huge gaps in the fossil record. By contrast, continual MCMC sampling of fossil ages from a prior distribution resulted in divergence time estimates that are in much better agreement with previous studies.

### Timetree Analyses With ILS and Hybridization

**Figure 5:**
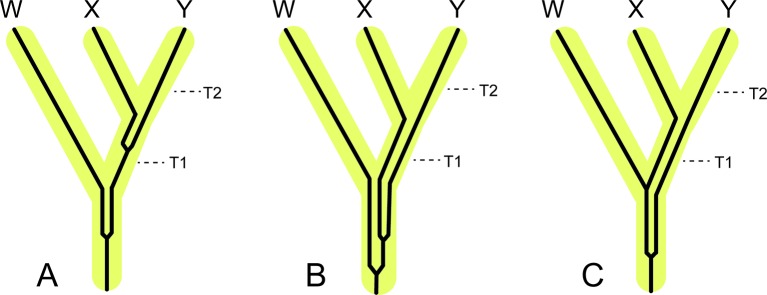
Examples of the effects of coalescence for individual genes on divergence time estimation relative to speciation times (i.e., incipient cladogenesis with cessation of gene flow), T1 and T2. Gene tree lineages are thin and black and are contained within thick and yellow species tree lineages. **(A)** Coalescence of a gene in the most recent common ancestor of X and Y and in the most recent common ancestor of X+Y and W. The topology of the species tree and the topology of the gene tree are congruent. Coalescence times for this gene exceed species divergence times, but by less than one internal branch. **(B)** Deep coalescence of a gene in the common ancestor of W, X, and Y in which the gene tree topology agrees with the species tree topology. **(C)** Deep coalescence of a gene in the common ancestor of W, X, and Y in which the gene tree topology conflicts with the species tree topology. All coalescences of genes in this figure are deeper than speciation times, so molecular clock estimates from these gene trees would be older than the true speciation times (T1 and T2).

**Figure 6:**
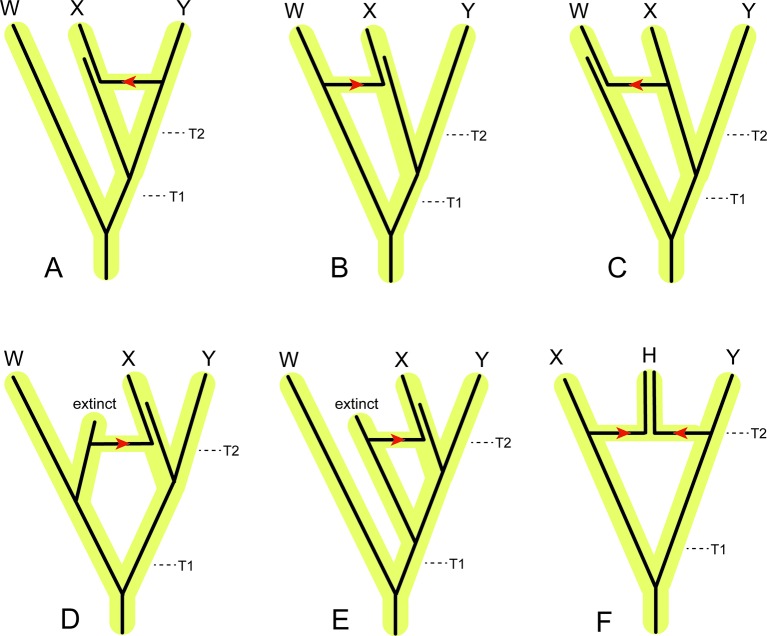
Examples of the effects of introgression/hybridization on divergence time estimation relative to speciation (incipient cladogenesis) times for individual gene segments that each have a single evolutionary history. **(A)** Introgression of a gene from the ancestor of Y to the ancestor of X. This gene flow pathway will decrease the estimated divergence time between X and Y relative to the actual speciation time (T2), but have no effect on the estimated divergence time between W and X+Y. **(B)** Introgression of a gene from the ancestor of W to the ancestor of X. This gene flow pathway will increase the amount of divergence between X and Y relative to the speciation time T2, and decrease the divergence between W and X+Y relative to the speciation time T1. If this gene flow pattern is pervasive through the genome, then the democratic vote (i.e., count of different genes supporting each topology) of traditional concatenation and coalescence methods will flip the topology to [(W,X),Y]. **(C)** Introgression of a gene from the ancestor of X to the ancestor of W. This gene flow pathway will have no effect on the estimated divergence between X and Y relative to the speciation time T2, but will decrease the estimated divergence between W and X+Y relative to the speciation time T1. If this gene flow pattern is pervasive through the genome, then the democratic vote will flip the topology to [(W,X),Y]. **(D)** Introgression of a gene from an extinct relative of W to the ancestor of X. Introgressed genes of this type will increase the estimated divergence between X and Y relative to the speciation time T2, and decrease the estimated divergence between W and X+Y relative to the speciation time T1. If this gene flow pattern is pervasive through the genome, then the democratic vote will flip the topology to [(W,X),Y]. **(E)** Introgression of a gene from an extinct relative of X+Y to the ancestor of X. Introgressed genes of this type will increase the estimated divergence between X and Y relative to the speciation time T2, but have no effect on the estimated divergence between W and X + Y relative to the speciation time T1. **(F)** Hybridization between the ancestors of X and Y results in a new species, (H), that coexists with the parental lineages that terminate in species X and Y. If the majority of the genome of H is derived from the ancestral lineage to X, then the democratic vote across the genome will favor the topology ((H,X),Y). Conversely, if the majority of the genome of H is derived from the ancestral lineage to Y, then the democratic vote across the genome will favor the topology [(H,Y),X].

Timetree analyses are aimed at estimating speciation times (incipient cladogenesis *sensu* de Queiroz, 1998), but timetree estimation is complicated by the observation that individual chromosomes and chromosomal segments may have different evolutionary histories. These differences can be the result of several processes, including coalescence (with or without ILS), recent and ancient gene flow, homoplasy, demography, natural selection, and sex-specific biases in gene flow. Each of these processes may differentially shape genetic variation within distinct, and non-random regions of a genome (Li et al., 2016; Li et al., 2019). Phylogenetic analysis of different genomic segments that are influenced by these different processes will yield trees with unique branching patterns and branch lengths, which when converted into time can produce a range of divergence estimates (Nachman and Payseur, 2012; Leaché et al., 2014; Fontaine et al., 2015). For example, coalescence will result in dates that are older than speciation times (**Figure 5**). In the absence of gene flow, coalescence times are expected to be older than speciation times even without ILS (**Figure 5A**). Under complete neutrality, the expected coalescence time for sequences that are sampled from individuals belonging to two different, completely isolated species is T + 2N, where T is the species divergence time and N is the population size of the ancestral species (Angelis and dos Reis, 2015). From this equation it is clear that expected coalescence times become increasingly older than actual speciation times with increasing ancestral population size. In some cases coalescence will not occur in the immediate common ancestor of two sister species and will occur even deeper in the tree, in which case it is referred to as deep coalescence (**Figures 5B, C**). In contrast, recent hybridization between sister taxa or the ancestors of extant sister taxa will result in divergence estimates between the hybridizing taxa that are younger than the initial time since divergence between the two lineages (**Figure 6A**). Hybridization between closely related non-sister taxa, including lineages that subsequently went extinct, can result in divergence time estimates between non-hybridizing taxa that are older than speciation times for some clades and younger for others (**Figures 6B–E**) (e.g., Figueiró et al., 2017; Barlow et al., 2018). Importantly, the direction of introgression has a critical role in altering divergence times for different clades (**Figure 6B** versus **Figure 6C**). Finally, hybridization may result in a new species that coexists with its parental lineages (**Figure 6F**). Hybrid speciation is rare in mammals, but hybrid origins have been suggested for the Neotropical bat *Artibeus schwartzi* (Larsen et al., 2010) and the Clymene dolphin (*Stenella clymene*) (Amaral et al., 2014).

Disregarding this potential variation in "gene" history across the genome can distort phylogenetic branch lengths and divergence estimates when multiple unlinked genomic loci are concatenated or combined in a coalescence analysis (Schierup and Hein, 2000; Posada and Crandall, 2002; Lemey and Posada, 2009; Li et al., 2019). Timetree methods such as *BEAST (Heled and Drummond, 2010) allow individual loci to have unique histories under the multispecies coalescent with ILS, but more complicated models that deal with ILS and introgression are still in their infancy, especially in cases where hybridization effectively overwhelms the phylogenetic signal of speciation across the majority of the genome.

### Hybridization

ILS has been widely recognized as a source of gene tree variation, and coalescent methods to accommodate this variation (together with other sources of variation) are becoming more widespread (e.g., Hobolth et al., 2007; Degnan and Rosenberg, 2009; Hobolth et al., 2011; Hibbins and Hahn, 2019; Bravo et al., 2019). However, most coalescence approaches assume all discordance among loci results from ILS (Liu et al., 2009), thus disregarding the potential contributions from post-speciation gene flow to phylogenomic discordance. The expansion of whole genome data has led to the recognition that interspecific hybridization is a widespread phenomenon across the tree of life and must be accounted for in phylogenomics and timetree estimation (e.g., Ai et al., 2015; Fontaine et al., 2015; Lamichhaney et al., 2015; Li et al., 2016; Martin and Jiggins, 2017; Árnason et al., 2018; Barlow et al., 2018; Palkopoulou et al., 2018; Li et al., 2019). A few cases in the literature (mosquitoes, butterflies, and cats) have shown that extensive hybridization can effectively replace the phylogenetic signal of original branching events across the majority of the genome, and in these instances the original signal for the deepest divergence point between taxa is only present in a minority of the genome (Fontaine et al., 2015; Edelman et al., 2019; Li et al., 2019). In such situations, traditional concatenation and coalescence approaches that use all of the data ("democratic vote" methods, Degnan and Rosenberg 2009) may fail to construct an accurate phylogeny of the original branching events, instead producing trees that reflect the most recent and extensive bouts of gene flow. These studies illustrate the importance of teasing apart segments of the genome that have different histories. We recommend that researchers examine X and Z chromosomal regions, especially low-recombination regions of these chromosomes, to determine if they record different histories than other regions of the genome. An additional point is that hybridization, if not accounted for, has the potential to result in zombie lineages where estimated divergence times are younger than minimum ages for speciation that are implied by the fossil record.

### Recombination

A second important observation from recent phylogenomic studies is that in lineages with an extensive history of hybridization and introgression, signatures of ancient (original) branching events are more rapidly depleted from chromosomal regions with high rates of meiotic recombination and more localized effects of linked selection (Brandvain et al., 2014; Schumer et al., 2018; Li et al., 2019; Martin et al., 2019). Conversely, regions of low recombination, particularly the X and Z sex chromosomes, are enriched for genomic segments that support the original species tree prior to reticulation (Fontaine et al., 2015; Edelman et al., 2019; Li et al., 2019; Martin et al., 2019). This enrichment on the sex chromosomes may be due to a higher density of reproductive isolating loci, reduced effective population size and hence reduced ILS, or some combination of these processes (Pease and Hahn, 2013; Presgraves, 2018). Perhaps paradoxically, Wang and Hahn (2018) showed that speciation genes, if they participate in Dobzhansky-Muller incompatibilities with other loci *via* epistatic interactions, are more likely to have gene trees that are discordant with the species tree.

Li et al. (2019) demonstrated a strong topological bias in high-recombination regions that are enriched for signatures of gene flow, supporting observations from previous simulation studies (Posada, 2000; Schierup and Hein, 2000; Leaché et al., 2014). The degree of branch length (and timetree) distortion is dependent on the temporal context and intensity of gene flow throughout the evolutionary history of the group (Li et al., 2019). Li et al. (2019) concluded that some star-like phylogenies could be artifacts of combining sequences derived from regions of the genome with elevated recombination rates and histories of gene flow, rather than accurate depictions of the diversification process. Collectively, these studies indicate that recombination rate is an important parameter to consider in phylogenetic inference and divergence time estimation (Edelman et al., 2019; Li et al., 2019; Martin et al., 2019). At the same time, recombination rate is a difficult parameter to include in most studies due to the rarity of recombination maps for most taxa. Yet this is likely to change in the near future as new linkage disequilibrium-based approaches allow for estimation of genome-wide recombination rates in a broader array of non-model organisms from population genomic sequence data (Stevison et al., 2016). Future studies should investigate the influence of local recombination rates and properly parsed out coalescence genes (e.g., Hobolth et al., 2007) on tree shape and divergence time estimation. One area of interest is to determine whether any of the previously supported models for mammalian evolution based on molecular studies are biased because of the distorting effects of combining loci from regions of the genome with highly variable or elevated recombination rates.

## Conclusions

The reconstruction of a reliable timetree for placental mammals is fundamentally important for understanding the potential role of the KPg extinction and other events in Earth history in promoting mammalian diversification. However, an agreed upon timetree remains elusive. Indeed, the number of models for placental diversification has increased, rather than decreased, over the last two decades. The list of competing models now includes the *Explosive*, *Soft Explosive*, *Trans-KPg*, *Long Fuse*, and *Short Fuse Models*. Unprecedentedly large phylogenomic and multigene data sets for placental mammals have become available in the last decade (Meredith et al., 2011; dos Reis et al., 2012; Song et al., 2012; dos Reis et al., 2014; Emerling et al., 2015; Foley et al., 2016; Tarver et al., 2016; Chen et al., 2017; Liu et al., 2017a; Wu et al., 2017). Similarly, O’Leary et al. (2013) and Halliday et al. (2019) have assembled the largest morphological data sets for Eutheria in this same time span. Molecular and morphological data can now be analyzed, either separately or in combination with each other, with increasingly complex approaches to timetree reconstruction (Kumar, 2005; Pyron, 2011; Ronquist et al., 2012; Heath et al., 2014; Kumar and Hedges, 2016) that have the potential to discriminate among competing models for placental diversification. However, some of the largest phylogenomic data sets have pervasive homology problems, often due to limitations of fragmented draft genome assemblies and their gene annotations, that limit their usefulness for phylogeny reconstruction and timetree estimation (Springer and Gatesy, 2016; Gatesy and Springer, 2017; Springer and Gatesy, 2018a; Springer and Gatesy, 2018b). Thus improving the contiguity and annotation of genome assemblies across the mammalian tree will reduce the probability of these artifacts. Similarly, new methods for timetree estimation have potential shortcomings that must be addressed if we are to reconstruct an accurate timetree for placental mammals. For example, tip dating methods employ morphological clock models that are conveniently borrowed from molecular evolutionary genetics, but these models may not be appropriate for morphological data. On the paleontological front, new fossil discoveries have the potential to provide decisive evidence for or against some of the models for placental diversification, but this requires that the fossils can be unambiguously placed in the eutherian tree. Variation in "gene" histories that results from the coalescent process (including ILS) and hybridization can distort phylogenetic branch lengths and divergence estimates when multiple unlinked genomic loci are combined together in a timetree analysis. The acquisition of high quality genomes for more and more mammalian taxa, combined with methods for detecting recombination and introgression, should help to facilitate the identification of genomic regions with different histories. The partitioning of the genome into these regions with different histories will be an important step in estimating species divergence times in the radiation of placental mammals. Finally, the acquisition of large-scale genomic data sets provides an opportunity for culling loci that exhibit a poor fit to models of sequence evolution. For example, an important conclusion from Liu et al. (2017a) is that suboptimal molecular clock loci and methods are a major cause of discordance among different studies that have investigated the timing of the placental radiation. A caveat here is that Liu et al.’s (2017a) results are also tainted by extensive homology problems and zombie lineages (Gatesy and Springer, 2017). Nevertheless, the important point here is that different models that are employed in timetree estimation, whether they be models of sequence evolution or models of rate variation across branches of a phylogenetic tree, should be adequate to describe the relevant process instead of just better fitting than other models. Timetree estimation is highly interdisciplinary, and we remain optimistic that improved estimates of the timing of the placental radiation will result from new fossil discoveries, additional high quality genomes, and improved models and methods for the analysis of these data.

## Data Availability Statement

Publicly available datasets were analyzed in this study. These data can be found here: Project DOI: 10.7934/P773, http://dx.doi.org/10.7934/P773.

## Author Contributions

MS, NF, JG, and WM conceived the study. PB performed pseudoextinction analyses. JG and MS collected data from genomic and fossil databases. MS, NF, JG, and WM wrote the manuscript. PB, NF, and JG constructed figures. PB provided comments on the draft manuscript. All authors read and approved the final draft for submission.

## Funding

This work was supported by National Science Foundation grant DEB-1457735 (JG and MS) and DEB-1753760 (WM).

## Conflict of Interest

The authors declare that the research was conducted in the absence of any commercial or financial relationships that could be construed as a potential conflict of interest.

The reviewer EG and handling Editor declared their shared affiliation at the time of review.
